# ﻿Taxonomic notes on the collection of the tribe Lamiini (Coleoptera, Cerambycidae) housed in the Natural History Museum, London

**DOI:** 10.3897/zookeys.1205.127164

**Published:** 2024-06-26

**Authors:** Guanglin Xie, Maxwell V. L. Barclay, Wenkai Wang

**Affiliations:** 1 Institute of Entomology, College of Agriculture, Yangtze University, Jingzhou, Hubei, 434025, China; 2 Department of Life Sciences, Natural History Museum, London, SW7 5BD, UK; 3 Hubei Engineering Research Center for Pest Forewarning and Management, Yangtze University, Jingzhou, Hubei, 434025, China

**Keywords:** Lamiinae, longhorned beetles, new synonym, taxonomy, type material, zoological collections

## Abstract

In the present paper, the genus *Mimomyagrus* Breuning, 1970 is synonymized with *Combe* Thomson, 1864, and *Mimomyagruspfanneri* Breuning, 1970 is considered a junior synonym of *Combebrianus* (White, 1858). The female of *Arctolamiasinica* Bi & Chen, 2022 is described for the first time and this species is reported as new to Myanmar. Type material of *Lamiapunctator* Fabricius, 1776 [= *Anoplophorachinensis* (Forster, 1771)], *Cerambyxgalloprovincialis* Olivier, 1800 [= *Monochamusgalloprovincialis* (Olivier, 1800)] and *Melanaustergranulipennis* Breuning, 1938 [= *Monochamusguerryi* Pic, 1903] are confirmed to be preserved in Natural History Museum, London.

## ﻿Introduction

As one of the largest natural history museums, the Natural History Museum, London [formerly British Museum, Natural History (BMNH), and hereafter NHMUK] houses abundant specimens of longhorn beetles from all over the world.

The first author had an opportunity to visit the NHMUK in 2022, and carried out research related to Cerambycidae for one year under the direction of the second author, Maxwell V. L. Barclay, the Senior Curator in Charge of Coleoptera at the NHMUK; the current paper is part of the research findings, together with four other separately published papers.

On the basis of the study of specimens of the tribe Lamiini preserved in the NHMUK, we propose a genus-level and a species-level new synonymy and confirm that the type material of three species, considered to be ‘lost’ and marked as ‘depositary not known’ (Tavakilian and Chevillotte 2023), are in fact deposited in the NHMUK.

## ﻿Material and methods

The material examined in this study is deposited in the NHMUK, and has already been identified by earlier specialists, except for the holotypes of *Combefulgurata* Thomson, 1864, *Mimomyagruspfanneri* Breuning, 1970 and *Combebrianus* m. *rufoantennatus* Breuning, 1954 used for comparison in this study, which are deposited in the Muséum national d’Histoire naturelle, Paris, Muséum cantonal des sciences naturelles, Lausanne and Royal Belgian Institute of natural sciences, Brussels, respectively.

The label text, which is reproduced verbatim without corrections or additions, is given in single quotation marks for all studied specimens. Individual labels are separated by a semicolon, and data on different rows by a single slash. Additional and explanatory comments by the authors are given in square brackets.

Photographs were taken using a Canon 7D Mark II DSLR camera with a Canon EFS 100 mm lens and edited using Adobe Photoshop 2020 release. Extended depth of field at magnifications was achieved by combining multiple images from a range of focal planes using Combine ZP or Helicon Focus software.

## ﻿Results

Based on our study on the tribe Lamiini preserved in the NHMUK, one genus-level synonym and one species-level synonym have been found, the female of one species is newly described with a new distribution record, and the type material of three species previously thought to be lost, has been confirmed to be preserved in the NHMUK. The details are as follows:

### ﻿Genus Combe

Taxon classificationAnimaliaColeopteraCerambycidae

Thomson, 1864

F87EA753-DC49-53B6-A5F0-27F9B1EA6B9A

Combe Thomson, 1864: 83; Pascoe 1866: 252; Lacordaire 1869: 344; Gemminger and Harold 1873: 3028; Aurivillius 1921: 119; Breuning 1961: 347. Type species:Combefulgurata Thomson, 1864 [= Combebrianus (White, 1858)].
Mimomyagrus Breuning, 1970: 88. Type species: Mimomyagruspfanneri Breuning, 1970. Syn. nov.

### ﻿ Combe brianus

Taxon classificationAnimaliaColeopteraCerambycidae

(White, 1858)

1EC0BCA7-46CF-5B30-A51C-76D65E81CA8B

Figs 1 , 2 , 3

Monohammus brianus White, 1858: 409. Type locality: Nepal.
Combe Fulgurata [sic] Thomson, 1864: 83. Type locality: unspecified.
Combe Brianus [sic]: Pascoe 1866: 244; Lacordaire 1869: 344; Gemminger and Harold 1873: 3028; Aurivillius 1921: 119.
Combe brianus : Breuning 1961: 347; Weigel 2006: 502; Barševskis 2018: 288, 2020: 178.
Combe brianus m. *rufoantennatus* Breuning, 1954: 7; 1961: 347. Type locality: Sumatra, Indonesia. Unavailable name.
Mimomyagrus pfanneri Breuning, 1970: 88. Type locality: Cameron highlands, Malaysia. Syn. nov.

#### Type material examined.

***Holotype*** of *Monohammusbrianus* White, 1858, female: ‘Type [p, label circular, red frame]; Brianus / n. o [h]; Mon. Brianus White / Nepal [h]; NHMUK013460997 [p]’; ***holotype*** of *Combefulgurata* Thomson, 1864, female: ‘HOLOTYPE [p, label rectangular, red]; Th. / TYPE [p, black frame]; Ex. Musaeo / JAMES THOMSON [p, black frame]; Ch. J. Gahan / vidit 1895. [p]; Combe / Brianus White / comp. with type / C. J. G. [h]; Brianus / White / Fulguratus / Type Thoms. / Malas. [h, label rectangular, red frame]; Muséum Paris / 1952 / coll. R. Oberthür [p]; HOLOTYPE / *Combe* / *fulgurata* Thomson, 1864 [p]; MNHN, Paris / EC23123 [p]’; ***holotype*** of *Combebrianus* m. *rufoantennatus* Breuning, 1954, female: ‘Holotype [p, label rectangular, red, black frame]; Solok / Sumatra / EX COLL. F. SCHNEIDER [p, black frame]; Coll. R. I. Sc. N. B. / Sumatra [p]; S. Breuning det., 195 [p] 4 [h] / Combe / brianus / rufoantennatus / mihi Typ [h]; L: Bull. Inst. r. sci. Nat. Belg. 1954, 30, 11: F. [h]’; ***holotype*** of *Mimomyagruspfanneri* Breuning, 1970, female: ‘TYPE [p,label rectangular, red]; Rég. Orientale / Malaisie [h] / Chassot [p] 7. 1968 [h]; GBIFCH / 00338292 [p]’; ***paratype*** of *Mimomyagruspfanneri* Breuning, 1970, female: ‘Para- / type [p, label circular, yellow frame]; PARATYPE [p, label rectangular, red]; Brit. Mus. / 19 [p] 77–313 [h]; Malaysia [p] / Cameron / h’lds iv/74 [h] / Coll. Pfanner [p]; *Mimomyagruspfanneri* Breuning [h]’.

#### Non-type material examined.

Thirteen specimens (5 males, 8 females) identified as ‘*Combebrianus*’ in NHMUK: Malaysia: 1 male: ‘MALAY PENIN: / Selangor. / Bukit Kutu / 3500 [h] ft. / 14 . 3. [h] 1931 [1 with handwriting] / H. M. Pendlebury. [p]; Ex Coll: / F. M. S. / Museum. [p, reverse side]; Ex F. M. S. Museum. B. M. 1955–354 [p, reverse side]] / NHMUK013461006 [p]’; 1 male: ‘Mal. / P. [h]; Pascoe / Coll. / 93–60 [p]; Combe / brianus / White [h, reverse side]; NHMUK013460999 [p]’; 1 male: ‘Penang [h, label circle]; Bowring. / 63. 47* [p] / Combe / brianus, White [h]; NHMUK013461002’; 1 male: ‘MALAY PENIN. / Kedah Perak. / 1000–2000 [h] ft. / 19 # March, 1928. [p]; Ex F. M. S. / Museum. / B. M. 1955–354 [p]; NHMUK 013461004 [p]’; 1 male: ‘MALAY PENIN [p] / Perak, F. M. S. / Maxwell Hill 3000 / June–July 1916 [h]; 332 [h]; Ex F. M. S. / Museum. / B. M. 1955–354. [p]; NHMUK013387052 [p]’; 1 female: ‘Rantau Panjang / Selangor. / H. C. Robinson. [p] / (12. 16 / V / 04) [h] / 1904–327.[p]; (4 / 1107)[p]; NHMUK013387035 [p]’; 1 female: ‘Malay / Penang [p]; Fry Coll. / 1905. 100. [p]; 17783 [h]; NHMUK013460998 [p]’; 1 female: ‘MALAY PENIN: [p] / Penang F. M. S. / Maxwell Hill 3000 / June–July 1916 [h]; 331 [h]; Ex F. M. S. / Museum. / B. M. 1955–354. [p]; Combe / Brianus, White / 2 [h]; NHMUK013387050 [p]’; 1 female: ‘Malay Penin / Panang, F. M. S. [p] j, / Gap / 2900 / May 1915 [h] 193 [p]2 [h] ; Ex F. M. S. / Museum. / B. M. 1955–354 [p]; NHMUK 013461000 [p]’; 1 female: ‘Malay / Penang [p]; Data unreliable / See Brit. Mus. / 1949–314. [p, label yellow]; Combe / brianus [h]; NHMUK013461003 [p]’; 1 female: ‘Malay / Penang / Batu Feringgi / catchment area [p] / 25 Aug 1963 [h] / H. T. Pagden; Pres by / Com Inst Ent / B. M. 1964-2 [p]; 119 [p]; Combe / brianus White [h] / E. A. J. Duffy det. 1964 [p]; NHMUK013461001 [p]’; 1 female: ‘MALAYA / Kuala Lumpur [p]/ Ampang Village [h] / Feb. 26^th^[h]19[p]35[h]; Ex F. M. S. / Museum. / B. M. 1955–354 [p]; NHMUK 013387047 [p]’;1 female: ‘MALAY PENIN: / Selangor. / Bukit Kutu / 3300–3500 [h] ft. / 14 . 3. [h] 1931. / H. M. Pendlebury. [p]; Ex Coll: / F. M. S. / Museum. [p, reverse side]; Ex F. M. S. Museum. M. 1955–354 [p, reverse side]; NHMUK013461005 [p]’.

**Figure 1.**
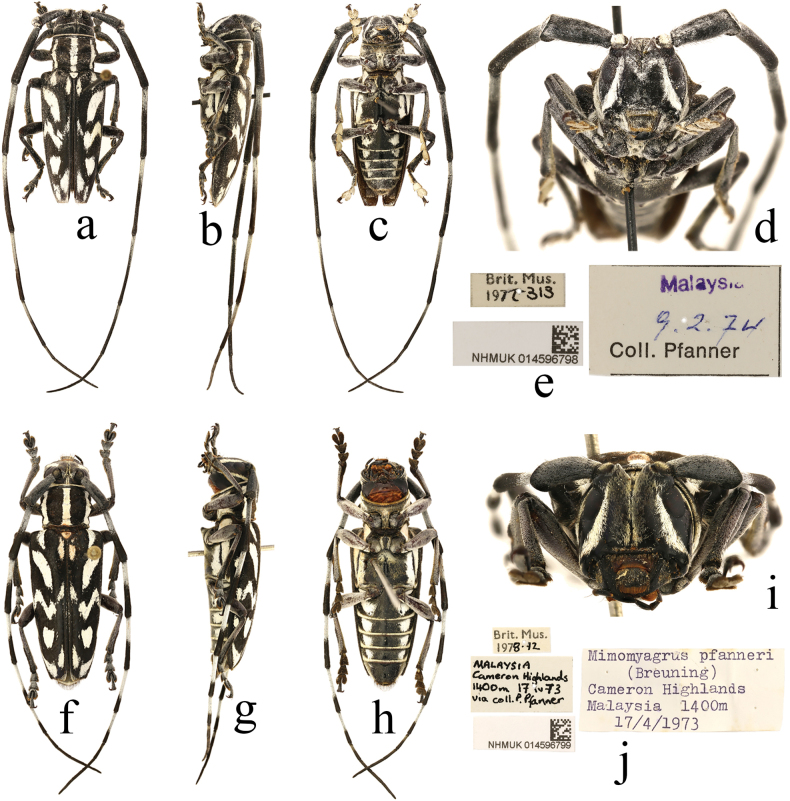
Habitus of *Combebrianus* (White, 1858) **a–d** male **f–i** female **e, j** labels.

Two specimens (1 male, 1 female) identified as ‘*Mimomyagruspfanneri* Breuning, 1970’ in NHMUK: Malaysia: 1 male (Fig. 1a–e): ‘Malaysia [p] / 9. 2. 74 [h] / Coll. Pfanner [p]; NHMUK014596798 [p]’; 1 female (Fig. 1f–j): ‘Malaysia / Cameron Highlands / 1400m 17 iv 73 / via coll. P. Pfanner [h]; Mimomyagruspfanneri / (Breuning) / Cameron Highlands / (Malaysia 1400 m / 17/4/1973) [p]; Brit. Mus. / 1978.72 [p]; NHMUK014596799 [p]’.

#### Comments.

**Figure 2.**
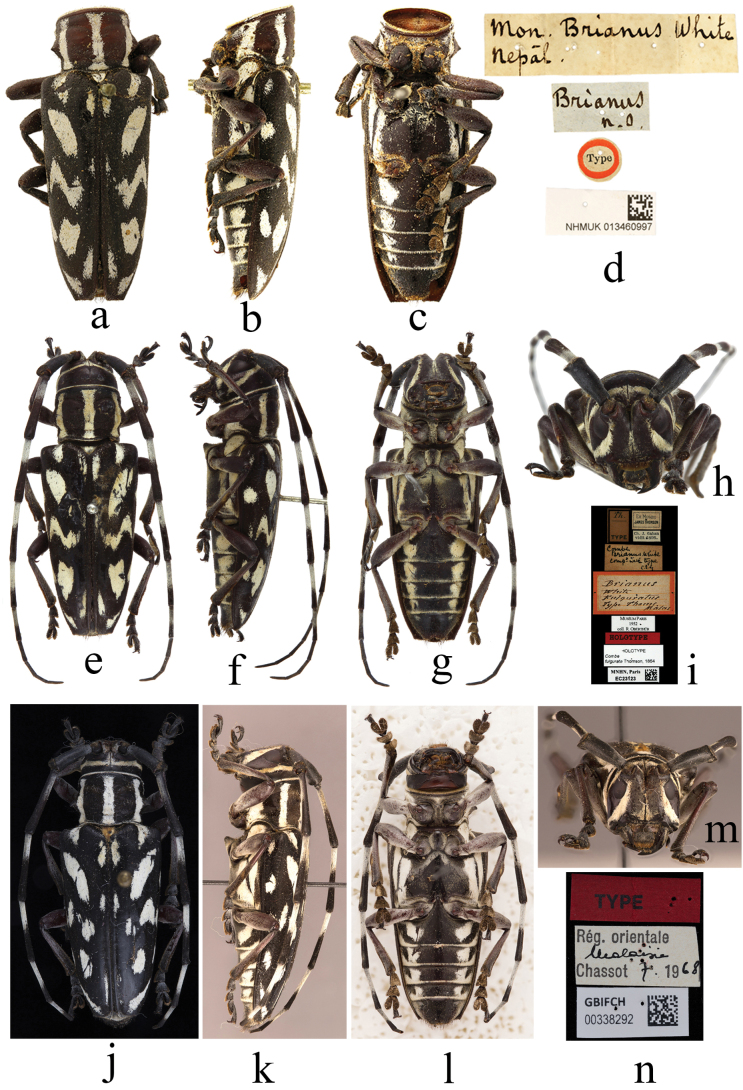
Habitus of *Combebrianus* (White, 1858) **a–c** holotype of *Monohammusbrianus* White, 1858, female **e–h** holotype of *Combefulgurata* Thomson, 1864, female **j–m** holotype of *Mimomyagruspfanneri* Breuning, 1970, female **d, i, n** labels.

White (1858) described *Monohammusbrianus* (Fig. 2a–d) based on a female specimen without head from Nepal. Thomson (1864) established the genus *Combe* for *Combefulgurata* (Fig. 2e–i) based on a female specimen without indicating locality. Pascoe (1866) transferred *Monohammusbrianus* to the genus.

**Figure 3.**
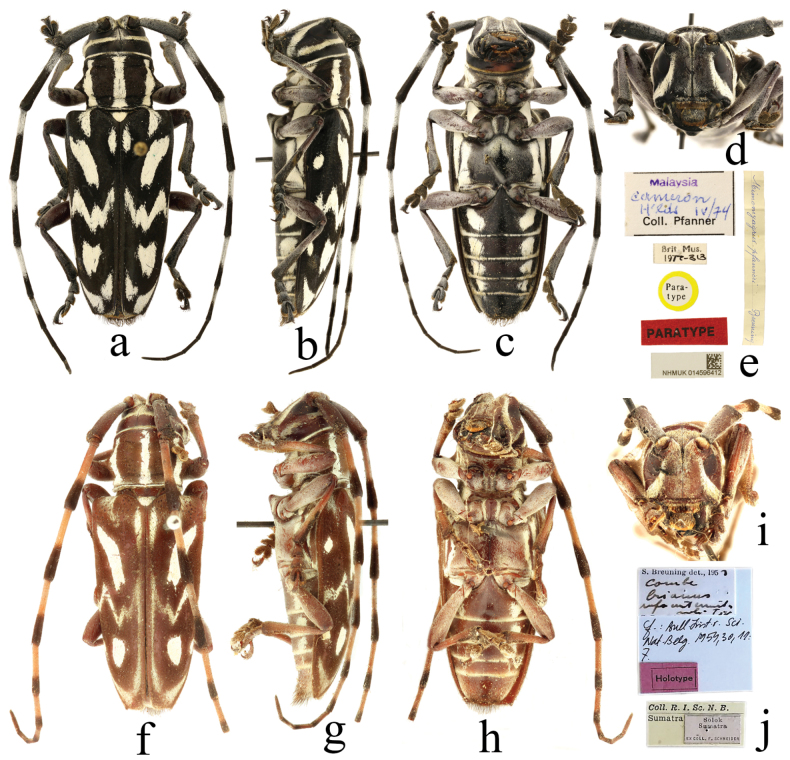
Habitus of *Combebrianus* (White, 1858) **a–d** paratype of *Mimomyagruspfanneri* Breuning, 1970, female **f–i** holotype of *Combebrianus* m. *rufoantennatus* Breuning, 1954, female **e, j** labels.

*Combe* and synonymized *Combefulgurata* with it based on the male and female specimens from Malacca, Malaysia. Breuning (1954) described a morph, *Combebrianus* m. *rufoantennatus* (Fig. 3f–j), based on a specimen of reddish-brown colour from Sumatra. Subsequently, Breuning (1970) described *Mimomyagruspfanneri* based on specimens from Cameron Highlands, Malaysia. Through studying these series of type and non-type specimens, we found that, in fact, they belong to the same species. Thus, here we propose the species *Mimomyagruspfanneri* as a junior synonym of *Combebrianus*.

Since *Mimomyagruspfanneri* is the type species of *Mimomyagrus*, this genus becomes a junior synonym of the genus *Combe*.

Breuning (1954) regarded the type specimen of *Combebrianus* m. *rufoantennatus* as a male. However, based on the ratio of the antennal length to body length, and the shape of the abdomen, it appears to be a female.

A questionable paratype of *Mimomyagruspfanneri* (Fig. 3a–e) is present in the collection of the NHMUK. According to the label, a handwritten collection date indicates that the specimen was collected in April, 1974 (Fig. 3e). The species was described in 1970 and the collection date was clearly recorded as ‘IV–V 1969’ in the original publication by Breuning, although on the label of the holotype a similarly handwritten date is given as ‘July 1968’. In any case, it is impossible for the collection date of the claimed paratype to appear after the publication date. Therefore, this specimen cannot be considered as a paratype.

### ﻿ Arctolamia sinica

Taxon classificationAnimaliaColeopteraCerambycidae

Bi & Chen, 2022

9C5492C3-8B44-52BB-94B2-3633FB0CC6F4

Figs 4a–j , 5f, i

Arctolamia sinica Bi & Chen, 2022: 199.

#### Description.

Female: similar to male, body length 33.5 mm, humeral width 12.0 mm. Body black, mostly densely clothed with reddish-brown pubescence, each puncture bearing a black or reddish-brown erect hair; glabrous areas showing black integument; elytra provided with five black pubescent patches. Frons, gena and vertex with black hairs, mouthparts with hairs lighter in colour, nearly yellowish-brown. Antennae with scape clothed with reddish-brown pubescence only on lateral margin; antennomeres III–V clothed with reddish-brown pubescence on basal half, antennomeres VI–VIII on basal half and antennomere XI on extreme apex clothed with greyish-yellow pubescence; antennomeres III–IV clothed with black pubescence on apical half, antennomeres V–VIII clothed with pubescence fading to chestnut on apical half; antennomeres IX–X and most of antennomere XI clothed with chestnut pubescence; scape and pedicel clothed with a black long hair on each puncture, denser on inferior margin; antennomeres III–VI fringed with long hairs below, reddish-brown on base and black on apex, antennomeres III–IV tufted with black hairs around apex. Pronotum clothed with black erect hairs on anterior, lateral and posterior margin, more on anterior margin; disc barely clothed with hairs on calli. Scutellum clothed with reddish-brown pubescence, without hairs. Elytra densely clothed with reddish-brown and black hairs, glabrous only on basal granules; each puncture bearing a long erect black or reddish-brown hair, black hairs short and stiff, reddish-brown hairs long and soft, arranged intermixed with each other; elytra with five black pubescent patches: a basal one located around scutellum, subangular; a lateral one on basal quarter after each humerus, smallest, oblique, not reaching the lateral margin; a large oblique one on each side behind the middle, not reaching suture and lateral margin. Underside densely clothed with reddish-brown pubescence, slightly greyish-yellow on mesosternum and mesoepisternum; ventrites furnished with greyish-yellow and black erect hairs. Legs mostly clothed with reddish-brown pubescence, tibiae and tarsi furnished with sparse black bristles.

Head sparsely punctate; frons transverse, slightly convex, with a distinct median sulcus extending to occiput. Eyes coarsely faceted, lower eye lobe transverse, about as long as gena; vertex uneven, with irregular wrinkles. Antennae distinctly shorter than body; antennal insertions conspicuously elevated, broadly separated; scape stout, gradually thickened apically, longest; antennomere III slightly shorter than antennomere IV, antennomere IV about as long as antennomere V, antennomeres V–X gradually shortened in length, antennomere XI about as long as antennomere VII. Pronotum transverse, with a pointed lateral spine on middle of each side; disc convex, coarsely rugose, with developed calli. Scutellum semicircular. Elytra broad, lateral margins gradually expanding outward at basal quarter after humeri, then convergent backward from middle to apices, apices conjointly rounded; about basal fourth provided the sparse, glabrous granules, of which several large granules regularly arranged in a row near the scutellum. Abdomen with first ventrite distinct longer than second and third ventrite, distal ventrite with apical centre slightly depressed, apical margin nearly straight. Legs moderately long and thick, metafemur reaching the middle of fourth ventrite.

#### Non-type material examined.

Myanmar: 1 male: ‘UPPER BURMA: / Nam Tamai Valley / 28. viii. 1938. / R. Kaulback. / B. M. 1938–741. [p]; Alt. 6,000 ft. / Lat. N. 27°42′. / Long. E. 97°54′. [p]; Arctolamia / fruhstorferi / Auriv [h] / DET. –E. F. GILMOUR [p]; NHMUK014596131’; 1 female: ‘UPPER BURMA: / Nam Tamai Valley / 28. viii. 1938. / R. Kaulback. / B. M. 1938–741. [p]; Alt. 6,000 ft. / Lat. N. 27°42′. / Long. E. 97°54′. [p]; NHMUK014596132’.

#### Comments.

**Figure 4.**
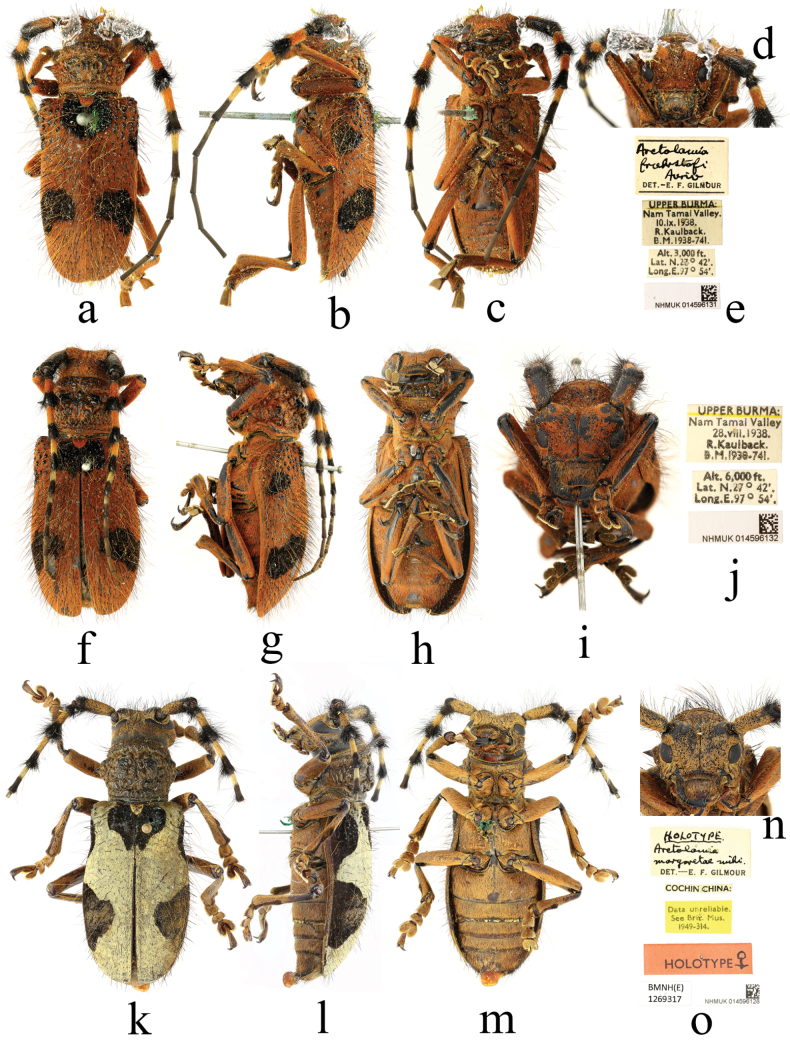
Habitus of *Arctolamia* spp. **a–j***Arctolamiasinica* Bi & Chen, 2022 **k–o** holotype of *Arctolamiamargaretae* Gilmour, 1950 **a–d** male **f–i, k–n** female **e, j, o** labels.

Gilmour (1950) misidentified the above pair of specimens as *Arctolamiafruhstorferi* Aurivillius, 1902 (Fig. 4a–j) and, using this as a comparison, described another species, *Arctolamiamargaretae* Gilmour, 1950 (Fig. 4k–o). In fact, *A.margaretae* is a junior synonym of *A.fruhstorferi*, as shown by Pu (1981), whereas the specimens misidentified by Gilmour as *A.fruhstorferi* represent *A.sinica* Bi & Chen, 2022.

Bi and Chen (2022) indicated that *A.sinica* can be differentiated from *A.fruhstorferi* by the absence of light-coloured pubescence on the dorsal surface of the scape and numerous large granules on the base of elytra. However, a male specimen of *A.sinica* from Myanmar shows the scape distinctly clothed with reddish-brown pubescence on dorsal surface (Fig. 4a, b, d) and there is a female specimen in the NHMUK identified as *A.fasciata* Gestro, 1891 that is actually supposed to be *A.fruhstorferi* (maybe it is a transitional form), which also has some large granules on the elytral base (Fig. 4a–e). This seems to imply that, given current knowledge, the main feature that distinguishes *A.sinica* from *A.fruhstorferi* is the reddish-brown pubescence on its body.

**Figure 5.**
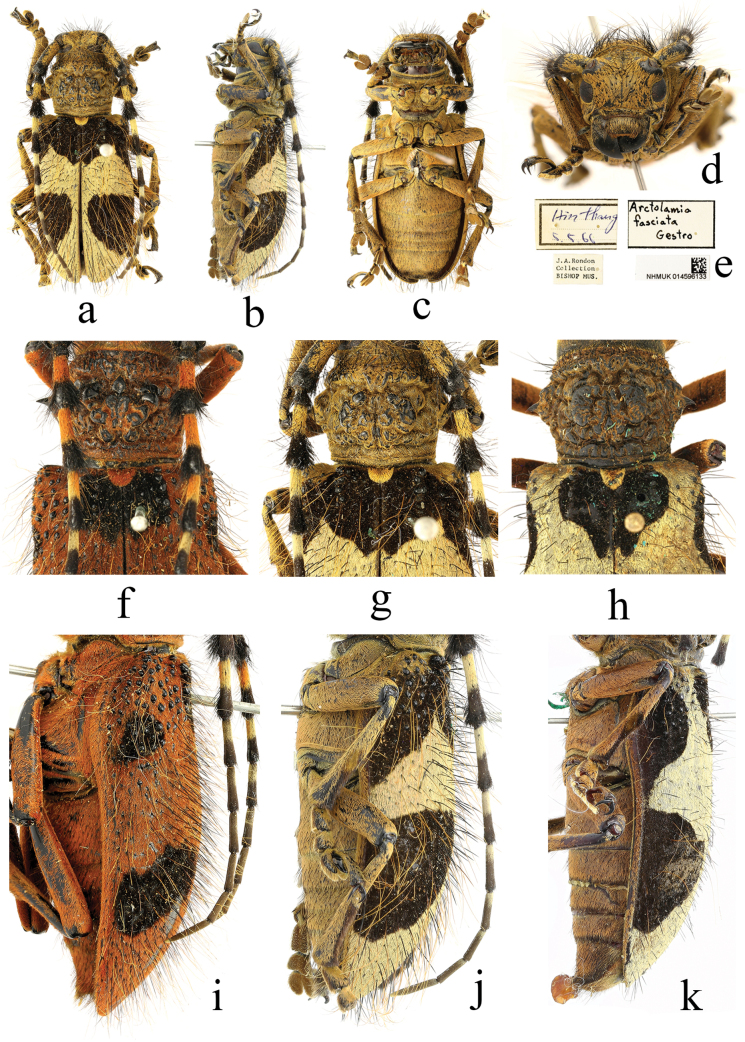
Habitus of *Arctolamia* spp. **a–d**, **g, j***Arctolamiafruhstorferi* Aurivillius, 1902 **f, i***Arctolamiasinica* Bi & Chen, 2022 **h, k** holotype of *Arctolamiamargaretae* Gilmour, 1950 **e** labels **a–d**, **f–k** female.

*Arctolamiasinica*, is also recorded in Myanmar for the first time based on the pair of specimens mentioned above.

##### ﻿Additional discoveries

**Figure 6.**
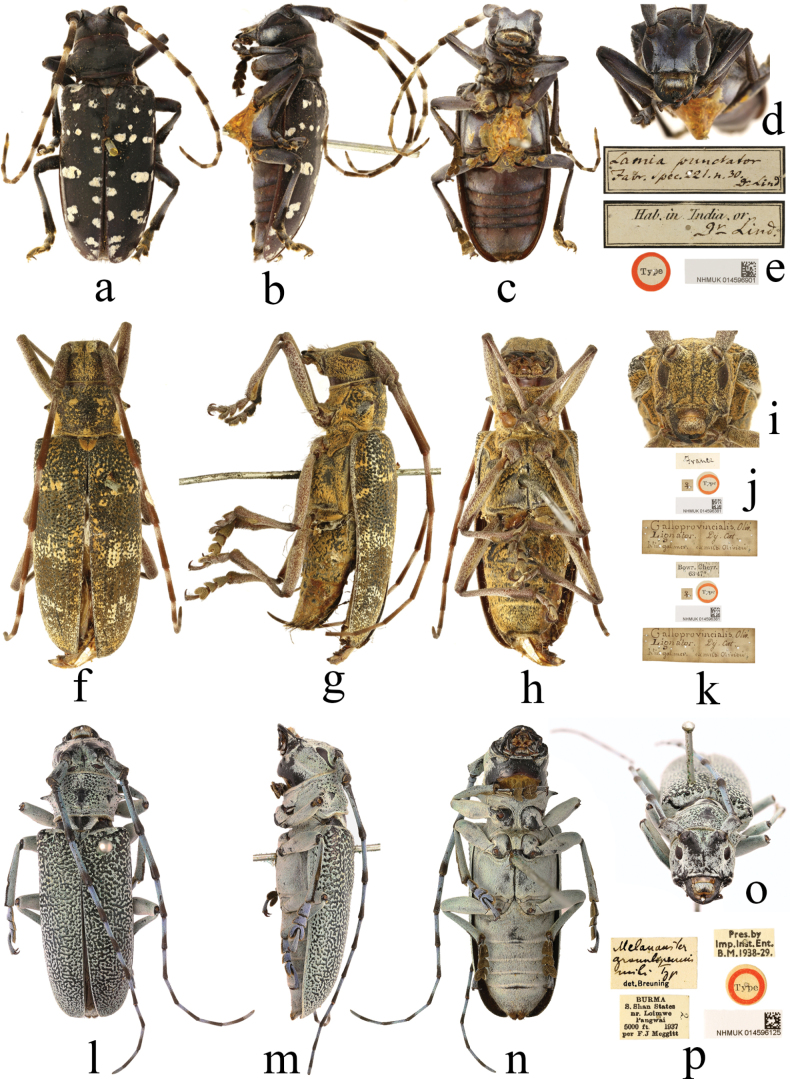
**a–d** type specimen of *Lamiapunctator* Fabricius, 1776, female **f–i** type specimen of *Cerambyxgalloprovincialis* Olivier, 1800, female **l–o** holotype of *Melanaustergranulipennis* Breuning, 1938, female **e, j, k, p** labels.

Type specimens of *Lamiapunctator* Fabricius, 1776 [= *Anoplophorachinensis* (Forster, 1771)] (Fig. 6a–e) and *Cerambyxgalloprovincialis* Olivier, 1800 [= *Monochamusgalloprovincialis* (Olivier, 1800)] (Fig. 6f–k) and the holotype of *Melanaustergranulipennis* Breuning, 1938 [= *Monochamusguerryi* Pic, 1903] (Fig. 6l–p) are confirmed to be preserved in the NHMUK; all were considered to be ‘lost’ and marked as ‘depositary not known’ (Tavakilian and Chevillotte 2023). *Lamiapunctator* Fabricius, 1776 and *Cerambyxgalloprovincialis* Olivier, 1800 are marked as having (missing) ‘holotypes’ by Tavakilian and Chevillotte (2023) and are labelled at the NHMUK with standard red framed ‘type’ discs, but there is no evidence of a holotype or of there being only one specimen in their original descriptions, so they are treated as syntypes. *Melanaustergranulipennis* Breuning, 1938 is described from a single female, and this specimen is a holotype.

## Supplementary Material

XML Treatment for Combe

XML Treatment for Combe brianus

XML Treatment for Arctolamia sinica

## ﻿Additional information

### Data availability

All of the data that support the findings of this study are available in the main text.
